# Congenital Intramuscular Cavernous Hemangioma: A Rare and Underreported Entity

**DOI:** 10.7759/cureus.99673

**Published:** 2025-12-19

**Authors:** Hari Vignesh, Pola Govardhan Kumar, Sundeep Selvamuthukumaran, Sreedevi B.V, Mahesh K.G

**Affiliations:** 1 General Surgery, Sree Balaji Medical College and Hospital, Chennai, IND; 2 General Surgery, SRM Medical College Hospital and Research Centre, Kanchipuram, IND

**Keywords:** benign mesenchymal tumors, cavernous sinus hemangioma, cavernous vascular lesion, hemangioma, intramembranous hemangioma

## Abstract

Hemangiomas are common pediatric vascular tumors characterized by endothelial cell proliferation. They usually appear shortly after birth and are more frequently observed in females. Deep-seated forms, such as cavernous hemangiomas, are more common, while those arising within muscle tissue are relatively rare. They are most commonly located in the head and neck. We present the case of a five-year-old female with a long-standing swelling in the left infra-axillary region. Biopsy confirmed an intramuscular cavernous hemangioma. This case highlights an uncommon anatomical location for this tumor type.

## Introduction

Hemangiomas are benign soft tissue tumors characterized by intimal proliferation of the veins [1]. Intramuscular hemangiomas (IMHs), a subtype of benign vascular neoplasms, most frequently occur in the trunk and extremities and account for less than 1% of all hemangiomas. They are classified into three types based on affected vessel size: capillary, cavernous, and compound [2]. Historically, cavernous hemangiomas present with complications such as hemorrhage, phlebolith formation, and pressure effects [3]. Treatment aims to halt growth, reduce volume, and promote regression [4]. Unlike malignant tumors, hemangiomas do not metastasize but undergo a natural course of proliferation followed by involution [5].

## Case presentation

A five-year-old female presented with a painless, nontraumatic swelling in the left infra-axillary region, measuring approximately 5 × 3 cm. The swelling was not associated with erythema, discharge, fever, or other signs of infection. She was fully immunized, had no known drug or food allergies, and her growth and developmental milestones were appropriate for her age. There was no significant medical or surgical history.

**Figure 1:**
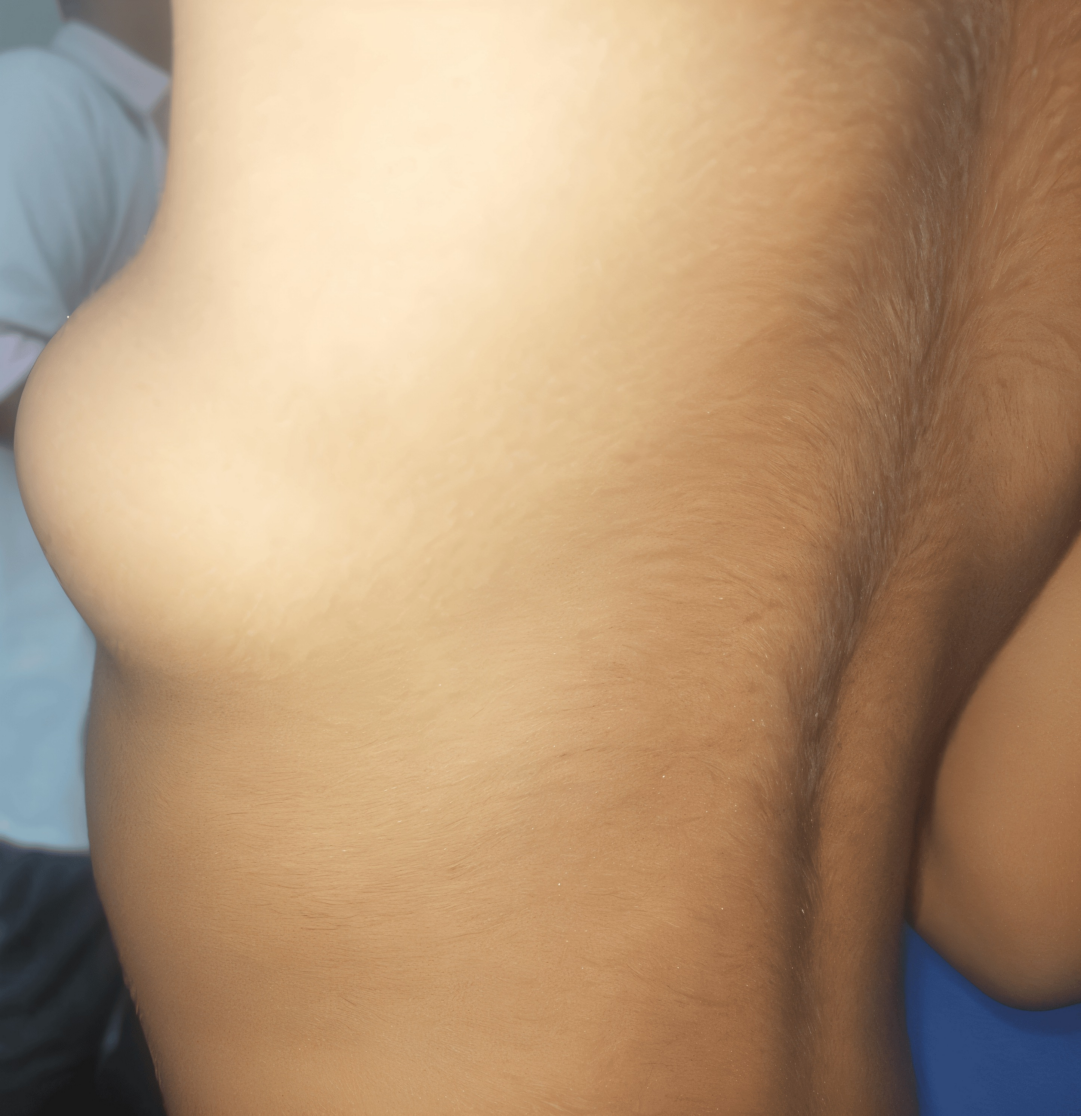
Swelling over the infra-axillary region

On physical examination, a solitary, well-circumscribed mass measuring approximately 5 × 3 cm was noted in the left infra-axillary region. The lesion had a smooth surface, was mildly compressible, and exhibited limited mobility in the horizontal plane while remaining fixed vertically. No overlying cutaneous changes were observed (Figure 1). There was no clinically significant restriction of arm movements.

**Figure 2:**
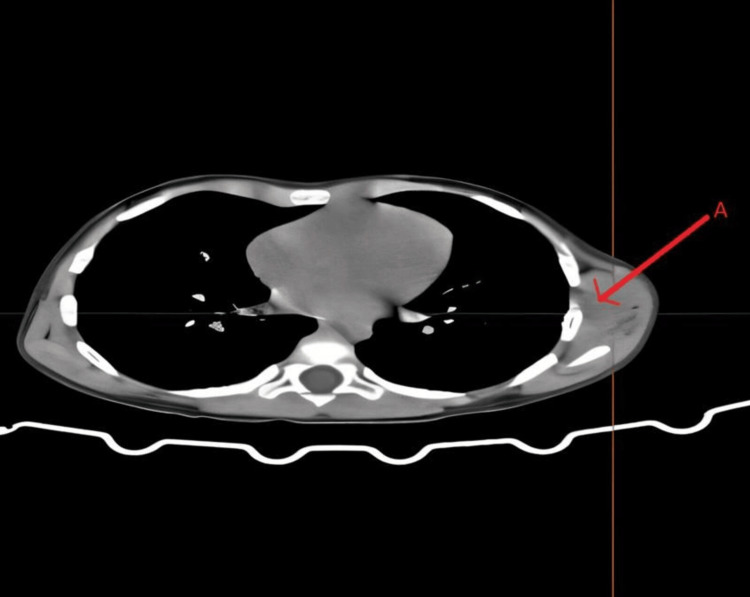
CT image showing a benign mesenchymal lesion, likely a hemangioma (A) A well-defined, ovoid mass lesion isodense to muscle is seen in the left posterolateral chest wall (red arrow), deep to the serratus anterior and overlying the costal muscles. The lesion shows no obvious invasion of the adjacent ribs or pleura. The overlying muscles show normal density.

A CT of the chest demonstrated a well-defined, loculated soft-tissue lesion measuring 5.0 × 2.0 × 3.8 cm within the deep intramuscular compartment of the left serratus anterior, overlying the mid-portions of the fifth to seventh ribs. Multiple punctate calcifications, possibly representing phleboliths, were identified within the lesion. These findings were suggestive of a benign mesenchymal neoplasm, most consistent with an IMH (Figure 2).

**Figure 3:**
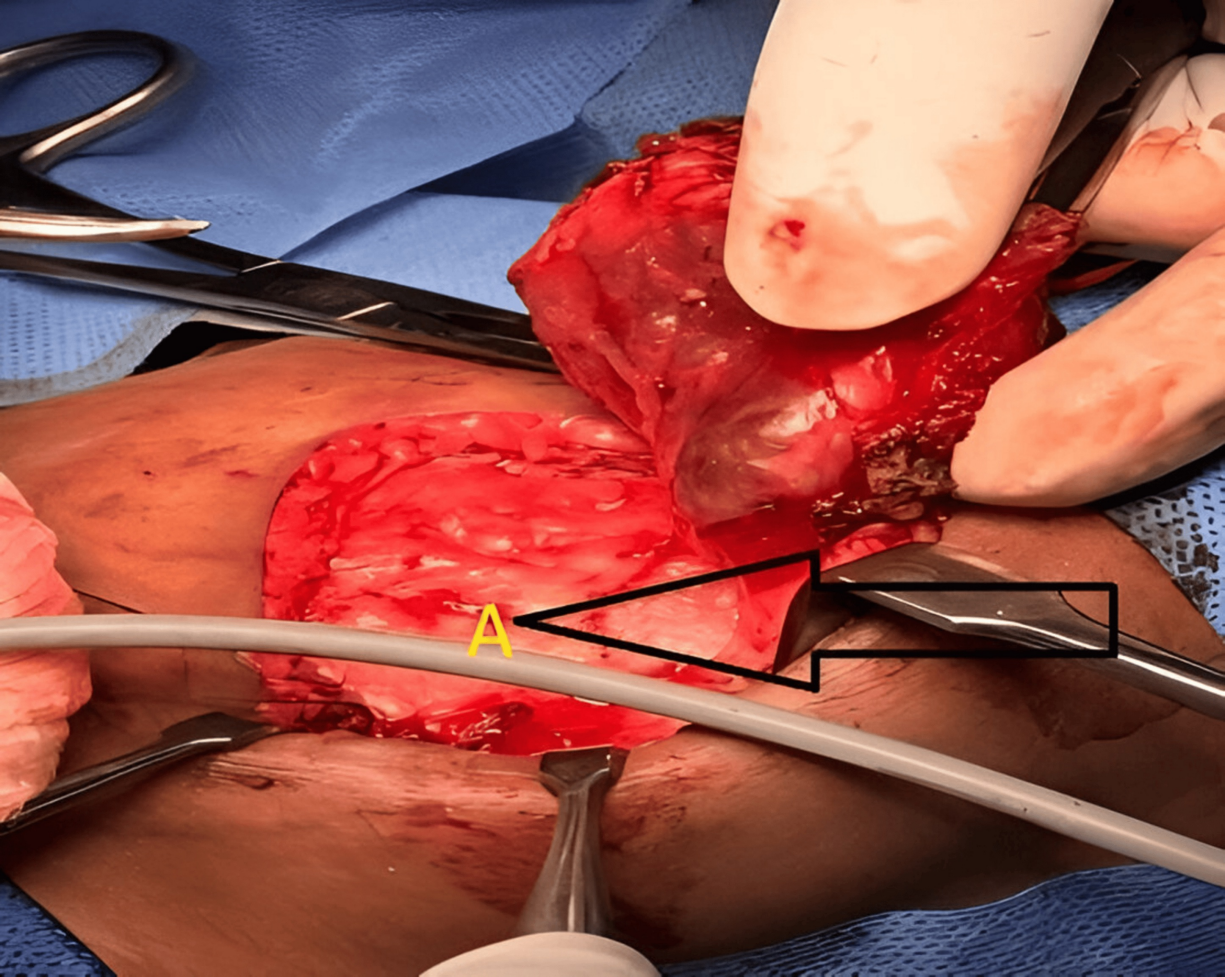
Superior pole of IMH attached to the base of the scapula (A) Intramuscular lesion within the serratus anterior muscle. IMH, intramuscular hemangioma

The patient underwent surgical excision (Figure 3).

**Figure 4:**
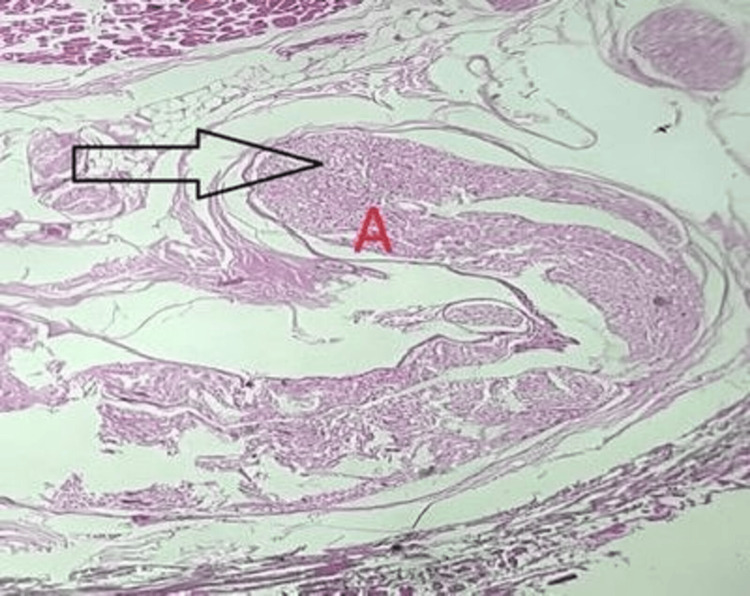
Lesion composed of dilated blood vessels of varying size with skeletal muscle bundles (high power, 100×) (A) Nerve bundle.

**Figure 5:**
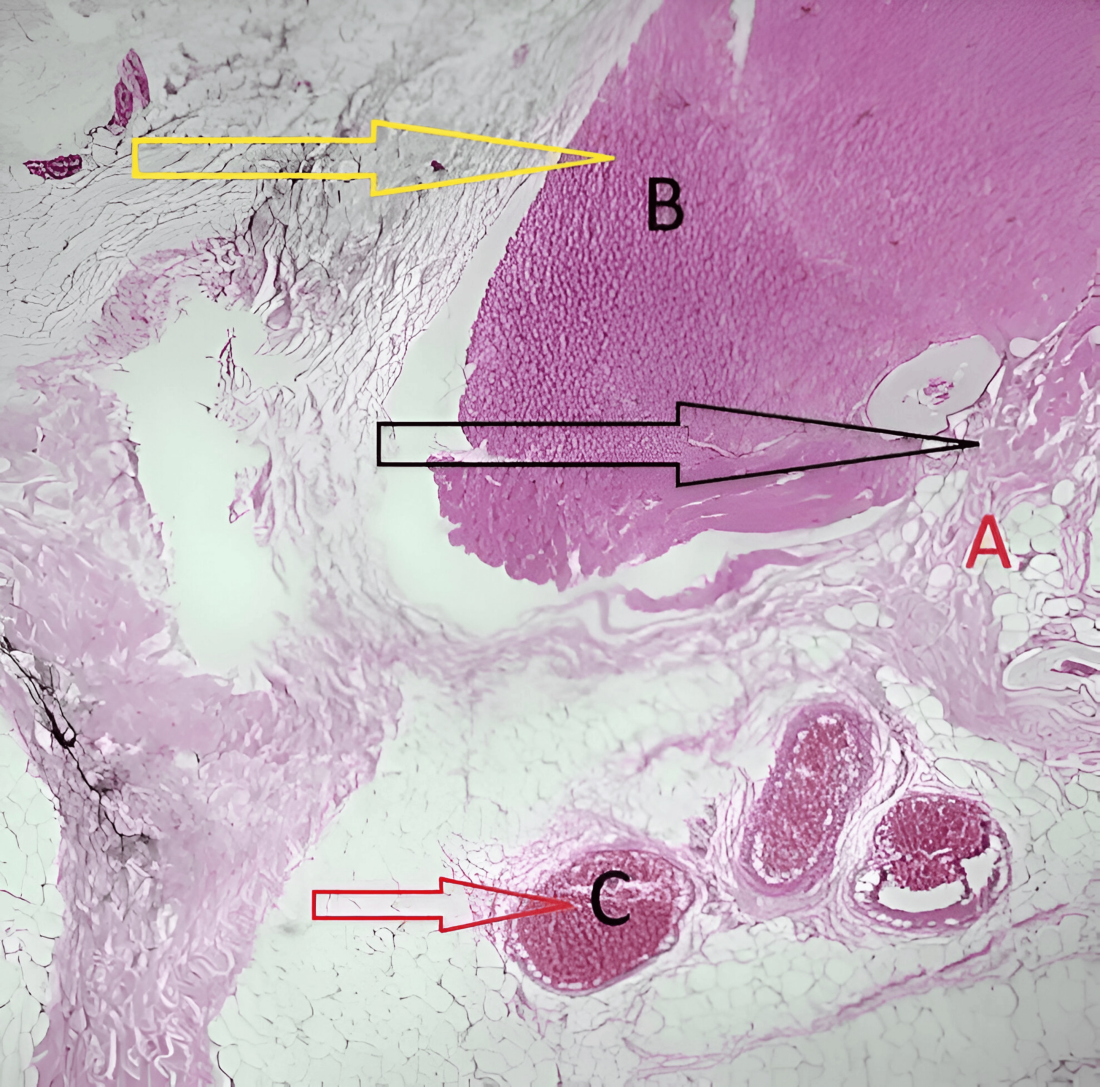
Lesion showing fibrocollagenous stroma with mature adipose tissue (low power, 40×; A-C) (A) Fibrocollagenous stroma with mature adipose tissue. (B) Skeletal muscle bundle. (C) Dilated blood vessel.

**Figure 6:**
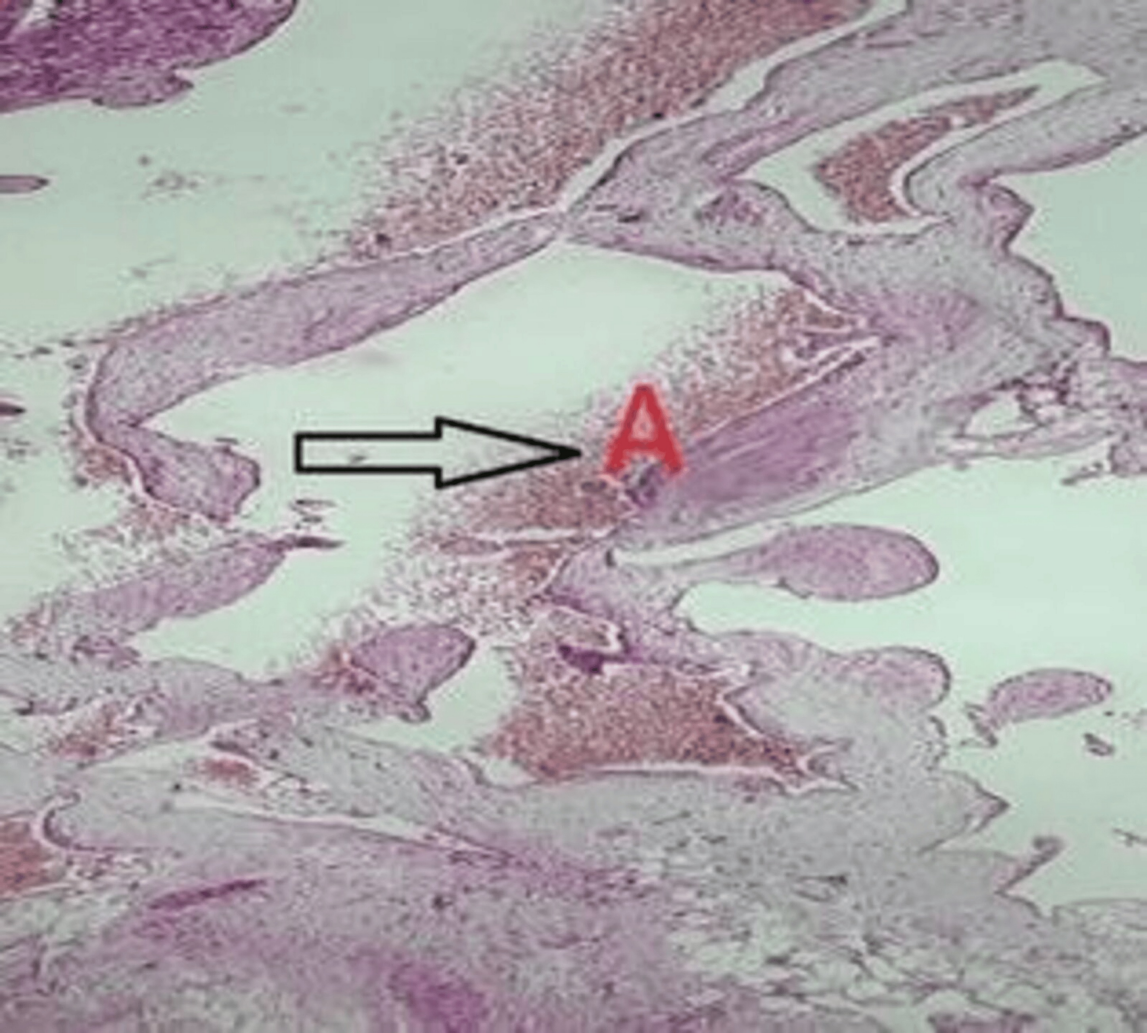
Slide showing dilated blood vessels filled with blood (low power, 40×) (A) Dilated blood vessel.

Histopathological examination revealed numerous dilated vascular channels of variable calibers embedded within a fibrocollagenous stroma, interspersed with skeletal muscle bundles and mature adipose tissue. Nerve fascicles and bony trabeculae were also present. These histological features were diagnostic of an intramuscular cavernous hemangioma (Figure 4, Figure 5, Figure 6).

## Discussion

IMHs are uncommon benign vascular tumors that arise within skeletal muscle and represent less than 1% of all hemangiomas [6,7]. They most frequently occur in the trunk and extremities, while chest wall involvement is distinctly rare. Because these lesions lie deep within muscle, they often present as slowly progressive, painless swellings without overlying skin changes, similar to the clinical features observed in our patient. Histopathology remains the definitive diagnostic method. Cavernous IMHs are characterized by dilated vascular channels lined by endothelial cells and interspersed with muscle fibers and fibrocollagenous stroma [8].

IMHs follow a benign natural history and do not undergo malignant transformation; many vascular tumors in infancy and childhood also show characteristic proliferative and involutional phases, as described in the classic classifications of pediatric vascular anomalies [9]. The clinical diagnosis of IMH can be challenging, as these tumors may resemble lipomas, fibromatosis, or soft-tissue sarcomas. Imaging, therefore, plays a central role. CT and MRI are the primary diagnostic tools. CT may reveal a well-defined soft-tissue lesion, and the presence of phleboliths is particularly suggestive of a cavernous hemangioma [10,11]. In this case, CT demonstrated a loculated intramuscular mass with punctate calcifications, consistent with IMH. The histologic findings in our patient aligned with these features.

Surgical excision is the preferred treatment for symptomatic, enlarging, or cosmetically concerning lesions. Complete removal is typically curative; however, recurrence rates of approximately 18-20% have been reported, mainly due to incomplete resection resulting from the infiltrative nature of these tumors within muscle planes [6]. Our patient underwent complete excision without complications. Early recognition and appropriate surgical management help prevent progressive enlargement, pain, or functional impairment. Given the rarity of IMHs in the chest wall, case reports such as this contribute valuable insight into their presentation, imaging characteristics, and optimal management.

## Conclusions

Intramuscular congenital cavernous hemangiomas are rare, benign vascular neoplasms arising within skeletal muscle. Although often present at birth, they may remain undiagnosed until later in childhood or adulthood due to their deep anatomical location and indolent progression. Diagnosis is based on clinical assessment and radiologic imaging, with histopathological confirmation required for definitive diagnosis. While nonmalignant, these lesions may cause pain, functional impairment, or cosmetic concerns, necessitating clinical intervention.

Surgical excision is the treatment of choice and is often curative; however, recurrence, reported at approximately 9%, is possible, particularly following incomplete resection. Early recognition and appropriate management are essential to minimize morbidity and optimize outcomes. Although congenital intramuscular cavernous hemangiomas are rare, they require prompt diagnosis and treatment to prevent significant morbidity. Complete surgical excision generally yields excellent outcomes. Given the rarity of this condition, further research is needed to better elucidate its pathogenesis and natural history.
